# Hypophosphatemia after intravenous ferric carboxymaltose in lung transplant recipients

**DOI:** 10.1007/s11739-026-04470-3

**Published:** 2026-07-24

**Authors:** Fabrizio Pumo, Noriane Andrina Sievi, Thomas Gaisl, Carolin Steinack, Lasse Marck, René Hage, Silvia Ulrich, Macé Schuurmans, Maurice Roeder

**Affiliations:** 1 Department of Pulmonology, University Hospital Zurich, Rämistrasse 100, 8091 Zurich, Switzerland; 2 Department of Endocrinology, Hospital Wetzikon, Wetzikon, Switzerland; 3 LungenZentrum Hirslanden Zurich, Zurich, Switzerland; 4 Medical Faculty, University of Zurich, Zurich, Switzerland

**Keywords:** Hypophosphatemia, Anemia, FCM, Lung transplantation

## Abstract

**Background:**

Intravenous ferric carboxymaltose (*FCM*) is frequently used to treat iron deficiency in lung transplant recipients (*LTRs*). Hypophosphatemia is a known adverse event after FCM administration, with reported significant hypophosphatemia following FCM administration in renal and heart transplant recipients. This study aimed to assess the incidence of hypophosphatemia after FCM administration and to identify potential risk factors in LTRs.

**Methods:**

This retrospective, single-center study included LTRs treated with FCM during post-transplant follow-up between 2015 and 2022 at the University Hospital Zurich. Patients were included if serum phosphate was measured within six months before and after FCM infusion. Phosphate levels and clinical data were extracted from electronic records, and hypophosphatemia was classified by severity. Changes in phosphate levels over time were analyzed using mixed-effects models, and multivariable logistic regression was used to identify predictors of hypophosphatemia. Of note, phosphate measurements were obtained at routine follow-up visits, with the first post-infusion measurement occurring at a median of 32 days.

**Results:**

Among 110 LTRs receiving FCM, 54.5% (*n* = 60) developed hypophosphatemia (≤ 0.81 mmol/L). Mild, moderate, and severe hypophosphatemia occurred in 23.6% (*n* = 26), 24.5% (*n* = 27), and 6.4% (*n* = 7) of patients, respectively. Use of 1000 mg FCM was associated with a higher risk of hypophosphatemia compared to 500 mg (OR (95% CI) of 2.54 (1.15–5.59)), increasing further after adjusting for baseline phosphate (OR (95% CI) of 5.18 (1.90–14.14)). Among patients with new-onset hypophosphatemia, 76% recovered within a median of 56 days. No serious clinical adverse events directly attributable to hypophosphatemia were documented.

**Conclusions:**

Hypophosphatemia frequently occurred after FCM infusion in LTRs, particularly following higher doses. Transplant physicians should be mindful of this risk and consider using individualized FCM doses or alternative intravenous iron formulations with lower hypophosphatemia risk, such as ferric derisomaltose in this population.

**Trial registration:**

www.ClinicalTrials.gov, NCT 06112236

## Background

Lung transplantation is associated with a wide range of complications, which are broadly categorized into infectious and non-infectious events. In addition to allograft-related complications such as primary graft dysfunction and chronic allograft dysfunction, anemia, metabolic, and renal disorders can also adversely affect the clinical course. Significant hypophosphatemia, found in 28% of patients following lung transplantation, has recently been reported; however, its underlying mechanisms and impact on clinical outcomes remain poorly understood [1]. Hypophosphatemia can cause general weakness, fatigue, bone and muscle pain, and in severe cases, osteomalacia with fractures [2]. These complications may hinder recovery and negatively impact outcomes following solid organ transplantation.

Dysregulated phosphate metabolism following kidney transplantation commonly leads to hypophosphatemia within the first three months post-transplantation. This is primarily due to persistently elevated levels of parathyroid hormone (*PTH*) and fibroblast growth factor 23 (FGF-23), which remain inappropriately high despite improved kidney function. The newly transplanted kidney, with its restored capacity to excrete phosphate, contributes to increased urinary phosphate losses. In addition to these hormonal drivers, several transplant-specific and general factors—including immunosuppressive therapy, chronic kidney disease (*CKD*), metabolic acidosis, persistent secondary hyperparathyroidism, and vitamin D deficiency, further contribute to phosphate wasting [3, 4].

Whether these factors also contribute to hypophosphatemia in recipients of other solid organ transplants remains unclear to date. Studies about phosphate metabolism in other solid organ transplant recipients are scarce. However, shared clinical factors such as similar immunosuppressive regimens or high incidence of CKD and vitamin D deficiency make it reasonable to assume that patients after heart, liver, or lung transplantation are also at increased risk for short- and long-term hypophosphatemia.

Moreover, post-transplant anemia is common among lung transplant recipients (*LTRs*), with iron deficiency being a frequent underlying cause [5]. The use of intravenous ferric carboxymaltose (*FCM*) is indicated for the treatment of iron-deficiency anemia when oral iron preparations are ineffective or not well tolerated. FCM is an iron complex that consists of a ferric hydroxide core stabilized by a carbohydrate shell that allows the controlled delivery of iron to the target tissue [6]. Common adverse events associated with FCM administration include headache, nausea, rash, and local injection-site reactions and hypophosphatemia is also frequently observed [6]. Although hypophosphatemia occurs also following intravenous ferric derisomaltose (*FDI*) and ferrumoxytol, the incidence of hypophosphatemia appears higher after FCM administration, the most commonly used intravenous iron compound in Switzerland. Systematic reviews and meta-analysis report pooled incidence of hypophosphatemia to be 47% (95% CI of 36–58%) after FCM and only 4% (95% CI of 2–5%) after FDI administration [7, 8]. The pathophysiological mechanisms behind hypophosphatemia after FCM is probably multifactorial and still under investigation. Following FCM administration, hypophosphatemia typically reaches its nadir within 1–3 weeks, with the peak occurring around day 14 after infusion [2, 9, 10]. While hypophosphatemia is generally transient and recovers over 8–10 weeks in most patients, persistent hypophosphatemia lasting 40–80 days or longer has been documented, particularly after repeated infusions [8, 11, 12]. The symptoms of hypophosphatemia, when present, are often nonspecific and may overlap with those of iron deficiency anemia itself, including fatigue and muscle weakness [9]. The risk of hypophosphatemia differs substantially between single and repeated FCM infusions, with recurrent dosing associated with higher rates of severe and prolonged hypophosphatemia and potential development of osteomalacia [7, 11]. After the administration of FCM, FGF-23 increases up to 3–6 fold. FGF-23 is a phosphate regulating peptide hormone secreted by osteocytes. FGF-23 increases urinary phosphate excretion by reducing proximal tubulus absorption of phosphate from the glomerular filtrate [2]. Moreover, FGF-23 suppresses 1-alpha hydroxylation inhibiting the activation of 25-(OH) vitamin D to 1,25-(OH)2 vitamin D (calcitriol). Also, the further metabolism to 24,25-(OH)2 vitamin D is increased by FGF-23 [13]. Reduced calcitriol levels cause hypocalcemia, which subsequently triggers an increase in circulating PTH. The phosphaturic effects of PTH aggravate the renal loss of phosphate. Additional factors beyond FGF-23 may contribute to or worsen hypophosphatemia following FCM administration. In particular, enhanced erythropoiesis following the normalization of iron stores can promote intracellular phosphate uptake, further lowering serum phosphate levels [14].

This study aimed to evaluate the impact of FCM infusion as a potential risk factor for the development of hypophosphatemia in LTRs, and to identify modifying clinical parameters in this population.

## Methods

### Study design and participants

This is a retrospective, single-centre study conducted at the Clinic of Pulmonology, University Hospital Zurich. The patient records of LTRs followed between January 2015 and December 2022 were screened for documented anemia, iron deficiency, and FCM infusion. LTRs were excluded from data analysis if general consent was absent or serum phosphate levels were not measured within 6 months before and after FCM infusion. Serum phosphate level is routinely determined in all regular outpatient follow-up visits of LTRs and life-long follow-up for all heath issues is performed and coordinated by the transplant pulmonologists. All LTRs treated with FCM infusion were included in the final analysis, and data before and after first FCM infusion were assessed. Demographic data, comorbidities, immunosuppressants, electrolyte levels, PTH, thyroid-stimulating hormone (*TSH*), kidney function, and phosphate influencing medications were extracted from the electronic patient data management program of the University Hospital Zurich.

The Ethics Committee of the Canton Zurich approved the study (BASEC-Nr 2022–02302) and the study was registered at www.ClinicalTrials.gov, NCT 06112236.

### Timing and indication for FCM administration

FCM was administered during the post-transplant follow-up period when iron deficiency anemia was identified, not as part of pre-transplant optimization. The median (quartiles) time from lung transplantation to FCM infusion was 54 (20–92) months. The decision to administer FCM was based on documented iron deficiency anemia with intolerance or inadequate response to oral iron supplementation.

### Measurements

LTRs undergo regular site visits after lung transplantation at the University Hospital Zurich following a standardized surveillance protocol: weekly visits during the first two months, biweekly for the next two months, monthly for the subsequent two months, and then every 8–12 weeks, adjusted based on clinical course. Phosphate measurements were obtained at routine follow-up visits, which occurred at variable intervals. The first follow-up measurement after FCM administration occurred at a median (quartiles) of 32 (18–55) days, with an overall range of 0–112 days. Data on demographics, comorbidities, medication, and iron deficiency treatment were assessed. Serum phosphate was assessed before FCM infusion and at multiple follow-up visits for up to 12 months thereafter. Other laboratory parameters, including ferritin, creatinine, PTH, and TSH, were extracted from routine clinical measurements before FCM administration when available in the electronic patient records. Hypophosphatemia was defined as mild (0.65−0.81 mmol/L), moderate (0.32−0.64 mmol/L) and severe (< 0.32 mmol/L). Multivariable regression analysis was used to investigate the association between hypophosphatemia and FCM administration.

### Data analysis and statistics

All data are presented as mean (standard deviation (*SD*)) or median (25%/75% quartiles) unless otherwise stated. Statistical analysis was performed with STATA 15.1 (StataCorp, Texas, USA). Variables were tested for normal distribution using Shapiro-Wilk test.

Statistical analyses included mixed effect regression model with random intercept and slope for each patient to assess overall changes in phosphate levels following FCM infusion, as well as subgroup analyses for the 500 mg and 1000 mg FCM doses separately. Dose-stratified analyses were restricted to patients receiving either 500 mg or 1000 mg FCM; three patients who received alternative FCM doses were excluded from these analyses. Chi-squared tests were used to compare the proportion of patients developing hypophosphatemia as well as severity proportions between the two dose groups. As a sensitivity analysis, we assessed whether the number of days between FCM administration and the follow-up visits influenced potential group difference in serum phosphate.

Additionally, a multivariable logistic regression model was employed to identify potential factors associated with the development of hypophosphatemia in LTRs receiving FCM, with an additional focus on whether FCM dose (500 vs 1000 mg) acted as a potential modifying factor. Potential predictors for hypophosphatemia were selected based on literature, data availability due to the retrospective study design, and adherence to model assumptions. Thus, baseline phosphate level, ferritin level at baseline, vitamin D dose, creatinine level at baseline, and the comparison of the commonly used immunosuppressant tacrolimus and cyclosporine were selected as potential predictors. Additional laboratory parameters potentially related to phosphate metabolism, including PTH and TSH, were collected when available and were reported descriptively. However, these variables were not included in the multivariable analyses because of substantial missing data, which would have considerably reduced the analyzable sample size and potentially resulted in unstable model estimates. To account for potential bias related to non-standardized follow-up timing, the interval between FCM administration and phosphate measurement (days) was additionally included as a covariate in all multivariable models.

A two-sided p-value of < 0.05 was considered as statistically significant.

## Results

### Study participants and baseline characteristics

A total of 110 LTRs underwent iron infusion with FCM. 48% were female and the median (quartiles) age at the time of FCM infusion was 62 (48/68) years. In our cohort, the most common disease leading to lung transplantation was chronic obstructive pulmonary disease and cystic fibrosis. The majority of patients were treated with triple immunosuppression comprising of calcineurin inhibitors (tacrolimus or cyclosporine), prednisone, and mycophenolate mofetil (*MMF*) or mycophenolic acid (*MPA*). 11 patients (10%) were treated with the mTOR inhibitor everolimus, either as a substitute for MMF/MPA or in addition to the established immunosuppressive therapy. 108 (98) and 92 (84%) LTRs used vitamin D and calcium supplementation with a median (quartiles) dose of 1600 (800/2800) IE/d and 1000 (800/1000) mg/d, respectively. 16 patients (15%) were treated with phosphate tablets. More than half of the patients (*n* = 61) received treatment with bisphosphonates. A summary of patient characteristics and further phosphate influencing medications are presented in table 1. Mean (SD) serum phosphate, median (quartiles) PTH, and median (quartiles) vitamin D levels were in normal range before FCM infusion with 1.06 (0.22) mmol/L, 72.6 (49.3/146.0) ng/L, and 32.5 (26.1/42.8) mcg/L, respectively. However, 16 LTRs showed mild (13%) to moderate (2%) hypophosphatemia at baseline, with no differences between the two dose groups (*p* = 0.358).

**Table 1:** Baseline characteristics

	*n* = 110
Age, years	62 (48/68)
Female, n (%)	53 (48.2)
Diagnosis leading to lung transplantation	
Interstitial lung disease, n (%)	22 (20.0)
Cystic fibrosis, n (%)	37 (33.6)
Chronic obstructive pulmonary disease, n (%)	38 (34.5)
Pulmonary Hypertension, n (%)	7 (6.4)
Others, n (%)	6 (5.5)
Comorbidities	
Chronic kidney disease, n (%)	71 (64.6)
Hyperparathyroidism, n (%)	15 (13.6)
Dialysis, n (%)	5 (4.6)
Osteopenia/osteoporosis, n (%)	95 (86.4)
Medication	
Calcineurin inhibitor, n (%)	106 (99)
Tacrolimus, n (%)	61 (55.5)
Cyclosporine, n (%)	49 (44.5)
MMF/MPS, n (%)	90 (84.1)
mTOR inhibitor, n (%)	11 (10.0)
Prednisone, n (%) Prednisone dose, mg/d	110 (100) 7.5 (6.3/7.5)
Vitamin D, n (%) Vitamin D dose, IE/d	108 (98.2) 1600 (800/2800)
Calcium supplementation, n (%) Calcium dose, mg/d	92 (83.6) 1000 (800/1000)
Rocatrol, n (%) Rocatrol dose, µg/d	7 (6.4) 0.25 (0.10)
Bisphosphonate, n (%)	61 (55.5)
Teriparatide, n (%)	1 (0.9)
Denosumab, n (%)	2 (1.8)
Diuretics, n (%)	39 (35.5)
Proton pump inhibitor, n (%)	108 (98.2)
Phosphate binder, n (%)	0 (0)
Phosphate supplementation, n (%) Phosphate dose, mg/d	16 (15) 192 (596)
Insulin, n (%)	35 (31.8)
Baseline blood values	
Phosphate level, mmol/L	1.06 (0.22)
25-Vitamin D, µg/L	32.5 (26.1/42.8)
Parathyroid hormone level, ng/L	72.6 (49.3/146.0)
Thyroid stimulating hormone level, mU/L	1.87 (1.10/2.55)

Values are mean (SD) or median (25%/75% quartiles) unless otherwise stated.

*MMF* mycophenolate mofetil, *MPS* = enteric-coated mycophenolate sodium

Of the 110 LTRs included in the study, 107 received either 500 mg or 1000 mg FCM and were therefore included in the dose-stratified analyses. Among these patients, 59 (55.1%) received 500 mg FCM and 48 (44.9%) received 1000 mg FCM intravenously. The remaining three patients received alternative FCM doses.

### Multiple FCM infusions and clinical outcomes

Among the 110 LTRs, 16 patients received more than one FCM infusion during the study period. No serious clinical adverse events directly attributable to hypophosphatemia, such as rhabdomyolysis, respiratory failure, or fractures, were documented during the follow-up period. However, given the nonspecific nature of hypophosphatemia symptoms and the retrospective design, mild symptoms such as fatigue or muscle weakness may have been underreported or attributed to other causes.

### Anemia associated blood values

Anemia before iron infusion was mild in our cohort with a mean (SD) hemoglobin level of 114.3 (18.6) g/L. The mean (SD) corpuscular hemoglobin (MCH) and corpuscular volume (MCV) of 30.1 (3.7) pg and 94.3 (9.2) fL defines the anemia as normochromic and normocytic anemia. The median (quartiles) ferritin level was low with 25 (16/42) mcg/L. Folic acid and vitamin B12 were in normal range. The median (quartiles) creatinine level was 100 (84/133) µmol/l. Table 2 details anemia-related values and their progression following FCM infusion.

**Table 2:** Anemia associated blood values

	Baseline	Follow-up
Ferritin, mcg/L	25 (16/42)	230 (100/427)
Hemoglobin, g/L	114.3 (18.6)	124.5 (15.9)
Mean corpuscular volume, fL	94.3 (9.2)	98.2 (8.8)
Mean corpuscular hemoglobin, pg	30.1 (3.7)	31.7 (3.4)
Folic acid, µg/L	7.6 (4.5/28.0)	–
Vitamin B12, ng/mL	407 (273/636)	–
Reticulocytes, G/L	54.7 (39.0)	–
Creatinine, µmol/L	100 (84/133)	–

Values are mean (SD) or median (25%/75% quartiles) unless otherwise stated

### Phosphate course after FCM infusion

**Fig. 1:**
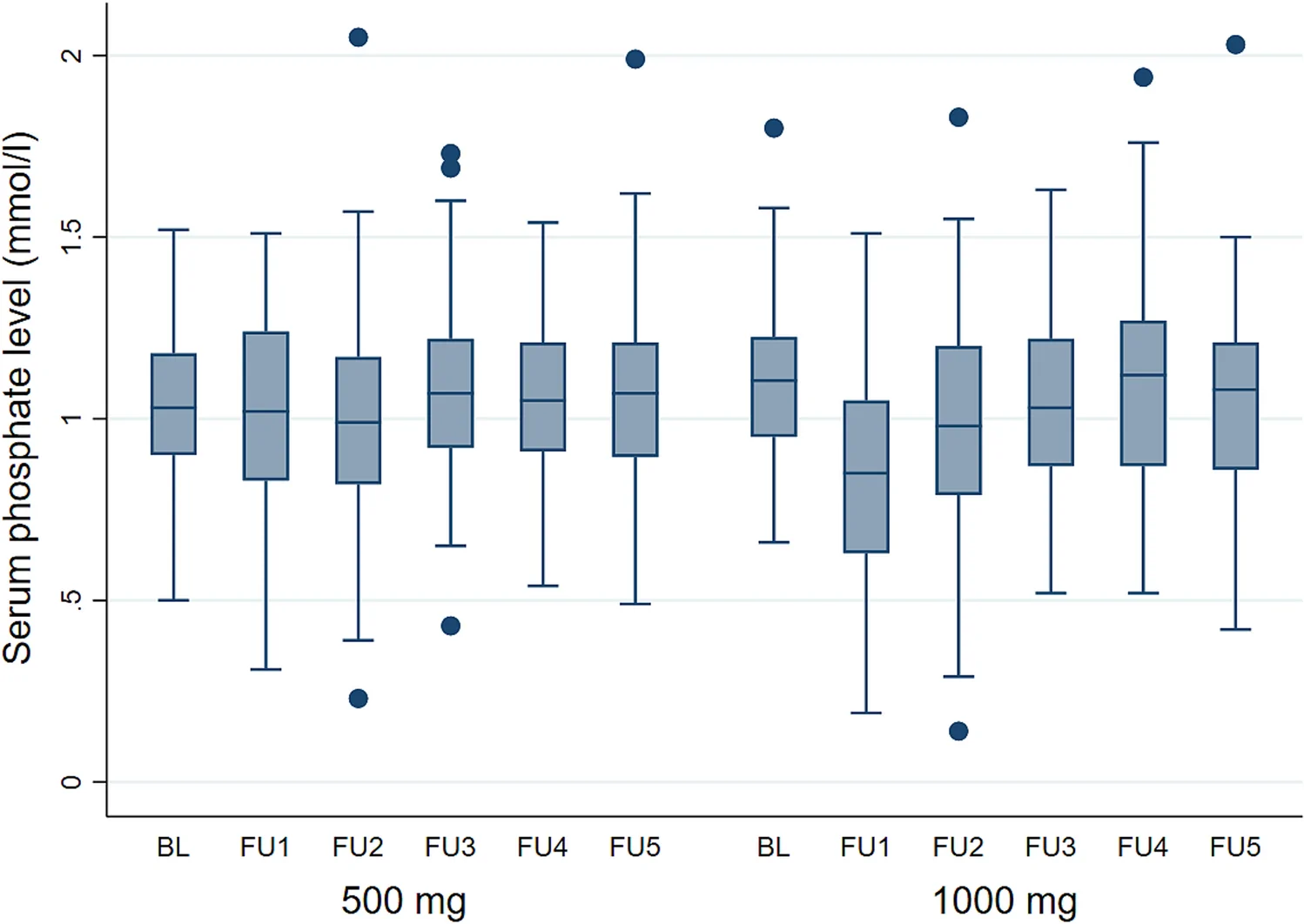
Serum phosphate level after the application of ferric carboxymaltose 500 mg or 1000 mg. *BL *baseline, *FU* follow-up, FU1–FU5 correspond to sequential follow-up visits occurring at a median (quartiles) of 32 (18–55), 63 (42–98), 102 (66–148), 132 (92–182), and 158 (117–233) days after FCM infusion, respectively

Of our cohort of 110 LTRs receiving FCM, 54.5% (*n* = 60) developed hypophosphatemia (serum phosphate ≤ 0.81 mmol/L). Mild hypophosphatemia (0.65−0.81 mmol/L) was described in 23.6% (*n* = 26), while moderate (0.32−0.64 mmol/L) or severe hypophosphatemia (< 0.32 mmol/L) was found in 24.5% (*n* = 27) and 6.4% (*n* = 7), respectively. After infusion of 1000 mg FCM, significantly more patients developed hypophosphatemia compared to infusion of 500 mg FCM (66.7% vs 44.1%, *p* = 0.020), with a higher number of patients with mild (31.3% vs 18.6%) and severe (10.4% vs 3.4%) hypophosphatemia after infusion of 1000 mg FCM. Fig. 1 shows the individual changes in hypophosphatemia severity from baseline to follow-up for both FCM doses. Patients with hypophosphatemia at baseline mostly remained hypophosphatemic, with half of patients increasing in severity.

**Fig. 2:**
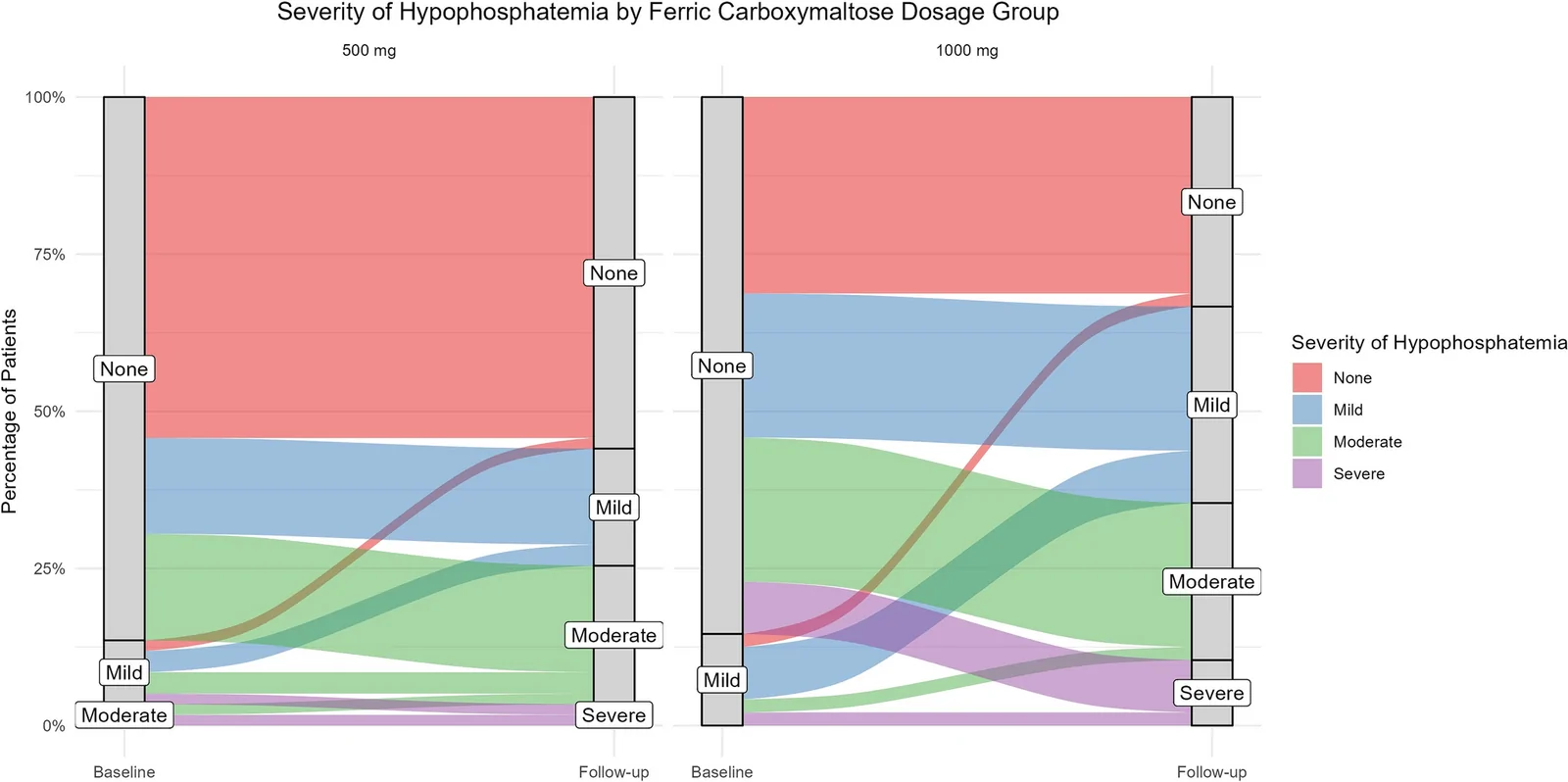
Alluvial diagram showing changes in hypophosphatemia severity categories (none, mild, moderate, severe) after administration of ferric carboxymaltose (*FCM*) 500 mg or 1000 mg. Abbreviations: *BL* baseline, *FU* follow-up, FU1–FU5 correspond to sequential follow-up visits occurring at a median (quartiles) of 32 (18–55), 63 (42–98), 102 (66–148), 132 (92–182), and 158 (117–233) days after FCM infusion, respectively

Figure. 2 and table 3 show the course of serum phosphate levels after the infusion of 500 mg and 1000 mg FCM, respectively, with an overall significant change over time. Mean (SD) maximal change in phosphate level was −0.29 (0.24) mmol/L. After the infusion of 1000 mg FCM, the phosphate level significantly declined with a mean (SD) maximal change of −0.37 (0.27), with the lowest mean phosphate level measured during the first follow-up visit (after a median of 32 days), while the infusion of 500 mg FCM showed no statistically significant decline with a mean (SD) maximal change of −0.21 (0.19). The difference in phosphate levels between treatment groups at follow-up 1 and 2 were not affected by the number of days between iron administration and the follow-up visit (*p* = 0.594 and *p* = 0.490, respectively).

**Table 3:** Course of serum phosphate level (mmol/L) at baseline and after infusion of ferric carboxymaltose (*FCM*)

	Baseline (*n* = 110)	First visit (*n* = 110)	Second visit (*n* = 110)	Third visit (*n* = 109)	Fourth visit (*n* = 105)	Fifth visit (*n* = 90)	p-value
Overall	1.06 (0.22)	0.92 (0.32)*	0.98 (0.33)*	1.06 (0.25)	1.06 (0.26)	1.06 (0.28)	0.005
FCM 500 mg (*n* = 59)	1.03 (0.21)	0.99 (0.30)	1.00 (0.33)	1.07 (0.24)	1.05 (0.24)	1.07 (0.26)	0.065
FCM 1000 mg (*n* = 48)	1.09 (0.23)	0.84 (0.33)*	0.98 (0.32)*	1.05 (0.26)	1.08 (0.29)	1.05 (0.30)	0.057
Days after Infusion	0	32 (18/55)	63 (42/98)	102 (66/148)	132 (92/182)	158 (117/233)	

Values are mean (SD) or median (25%/75% quartiles). *significant change from baseline. Data are presented for patients with available phosphate measurements at each time point. The difference in sample size between the dose groups (*n* = 59 for 500 mg and *n* = 48 for 1000 mg; total *n* = 107) and the overall cohort (*n* = 110) reflects three patients who received FCM doses other than 500 mg or 1000 mg and were therefore excluded from the dose-stratified analyses

Of 46 patients with normal phosphate level at baseline and hypophosphatemia at follow-up, 35 patients (76%) recovered during follow-up period after a median (quartile) duration of 56 (28/92) days. There was no statistical difference in recovery time between the two dose groups with median (quartile) duration of 59 (36/92) days after infusion of 500 mg FCM vs 51 (28/70) days after 1000 mg FCM.

### Predictors of hypophosphatemia after FCM infusion

The dose of FCM is a significant predictor of hypophosphatemia after FCM administration. The infusion of 1000 mg FCM instead of 500 mg doubles the risk for hypophosphatemia (OR (95% CI) 2.54 (1.15−5.59), p < 0.001). This risk is even higher after adjustment for phosphate baseline level (OR (95% CI) 5.18 (1.90−14.14), p < 0.001). Patients receiving the higher FCM dose had significantly higher odds of developing mild hypophosphatemia (OR (95% CI) of 2.37 (1.62−3.48), p < 0.001) and severe hypophosphatemia (OR (95% CI) of 3.31 (1.66−6.60), p < 0.001) compared to those receiving the lower dose, after adjusting for baseline phosphate levels. The odds of moderate hypophosphatemia after adjusting for baseline phosphate level was comparable between the two doses groups (OR (95% CI) of 1.34 (0.92−1.96), *p* = 0.126). Multivariable logistic regression analysis was performed to investigate potential effects of predefined variables on the development of hypophosphatemia in LTRs receiving FCM administration, adjusted for time between FCM administration and phosphate measurement (table 4). Higher phosphate baseline levels were highly negatively associated with hypophosphatemia after FCM infusion (OR (95% CI) 0.006 (0.000−0.091)). In contrast, baseline ferritin level, vitamin D dose, baseline creatinine level, immunosuppressive treatment (tacrolimus vs cyclosporine), and bisphosphonate use were not significantly associated with the development of hypophosphatemia. Moreover, dose of FCM administration did not modify potential effects of predefined variables on the development of hypophosphatemia (table 5).

**Table 4:** Multivariable regression analysis of possible predictors of risk of hypophosphatemia after infusion of ferric carboxymaltose (*FCM*)

Variable	Odds ratio	95 % Confidence interval	p-value
Phosphate level baseline, mmol/L	0.006	0.000−0.091	<0.001
Days from FCM infusion to phosphate measurement, days	0.98	0.96−1.00	0.061
Ferritin level baseline, µg/L	1.00	0.99−1.01	0.842
Vitamin D dosage, IE	1.00	1.00−1.00	0.216
Creatinine level baseline, µmol/L	1.00	0.99−1.01	0.372
Tacrolimus vs. Cyclosporine (reference) use	2.04	0.75−5.58	0.163
Bisphosphonate use	1.11	0.43−2.84	0.833

**Table 5:** Multivariable logistic regression analysis evaluating interaction between ferric carboxymaltose (*FCM*) dose and potential predictors of hypophosphatemia, adjusted for time from FCM infusion to phosphate measurement

Variable	Odds ratio	95% Confidence interval	p-value
Phosphate level baseline, mmol/L	0.00	0.00−237.9	0.320
Ferritin level baseline, µg/L	0.99	0.95−1.03	0.614
Vitamin D dosage, IE	1.00	1.00−1.00	0.332
Creatinine level baseline, µmol/L	0.99	0.96−1.02	0.451
Tacrolimus vs. Cyclosporine (reference) use	3.29	0.24−44.53	0.370
Bisphosphonate use	6.60	0.49−89.12	0.155
Time from FCM infusion to phosphate measurement, days	0.99	0.96−1.02	0.381

## Discussion

LTRs face various long-term complications beyond graft dysfunction, including metabolic imbalances like hypophosphatemia. While its exact cause and significance are not fully understood, hypophosphatemia can result in fatigue, muscle weakness, and bone pain, potentially delaying recovery and impacting overall outcomes [2].

This retrospective, observational cohort study examined the association between FCM administration and hypophosphatemia, revealing that 54.5% developed hypophosphatemia, particularly following higher doses of FCM.

Hypophosphatemia is a well-known side effect of FCM infusion in non-transplant patients. A comprehensive post-hoc analysis of 45 interventional trials reported hypophosphatemia (defined as serum phosphate < 0.81 mmol/L) in 41.4% of all participants, with severe hypophosphatemia (< 0.3 mmol/L) occurring in 0.7% of cases [7]. A recent meta-analysis including prospective clinical trials reported a pooled incidence of hypophosphatemia of 47% (95% CI of 36–58) after FCM administration [8].

The incidence of hypophosphatemia in our cohort (54.5%) was comparable to previously reported pooled estimates and highlights that LTRs are similarly affected by FCM-induced phosphate disturbances.

Sari et al. investigated the effects of FCM in 23 kidney transplant recipients and observed hypophosphatemia in 56.5% of patients; however, none developed serum phosphate levels below 0.3 mmol/L [15]. In a post-hoc analysis involving 39 heart transplant patients, 25.6% developed hypophosphatemia, defined by a serum phosphate threshold of < 0.85 mmol/L. In this cohort as well, the incidence of severe hypophosphatemia among transplant patients was low with only three heart transplant recipients showing symptoms or phosphate levels below 0.5 mmol/L [16]. The proportion of LTRs developing severe hypophosphatemia (6.4%) was slightly lower in our cohort than that reported in other studies including various patients with iron-deficiency anemia (8.8–11.3%), although it remains clinically relevant given the known risks associated with severe phosphate depletion [8, 17, 18].

The pathophysiology of FCM-induced hypophosphatemia in LTRs likely involves a complex interplay between FCM-driven FGF–23 elevation and the pre-existing disturbances in mineral metabolism characteristic of the post-transplant state. FCM administration triggers a marked increase in intact FGF–23, which promotes renal phosphate wasting through downregulation of sodium-phosphate cotransporters in the proximal tubule and simultaneously suppresses 1,25–(OH)₂ vitamin D synthesis [2, 12]. In LTRs, this acute FGF–23-mediated phosphaturia is superimposed on a background of chronic metabolic derangements: 64.6% of our cohort had CKD, 13.6% had documented hyperparathyroidism, and 86.4% had osteopenia or osteoporosis. CKD in the post-transplant setting creates a paradoxical situation with respect to phosphate handling. On one hand, reduced glomerular filtration rate may attenuate the phosphaturic response to FGF–23, potentially explaining the slightly lower rate of severe hypophosphatemia in our cohort (6.4%) compared to non-CKD populations (8.8–11.3%) [8, 17, 19]. This is further corroborated by Schaefer et al [8]., who demonstrated significantly lower hypophosphatemia rates and higher phosphate levels in patients with CKD compared to those with preserved renal function. On the other hand, calcineurin inhibitor–induced tubular dysfunction, persistent secondary hyperparathyroidism, and vitamin D deficiency—all common among LTRs—may also contribute to phosphate wasting through PTH-mediated mechanisms that could operate independently of, or in parallel with, the FGF-23 pathway [3, 4, 17, 19]. Consequently, serum phosphate levels may reflect the interplay between reduced glomerular filtration, which tends to preserve phosphate, and increased tubular phosphate excretion, which may promote phosphate loss. This complexity may explain why baseline creatinine alone did not predict hypophosphatemia in our multivariable model, whereas baseline phosphate level, which integrates all of these competing influences, was the strongest predictor.

Encouragingly, 76% of patients with new-onset hypophosphatemia recovered within a median of 56 days, suggesting transient phosphate depletion in most cases. This is in line with findings from Fragkos et al [18]., where 35% had persistent hypophosphatemia for up to 90 days. However, it is noteworthy that approximately one-third of affected LTRs showed persistent hypophosphatemia beyond this period, indicating that long-term disturbances in phosphate homeostasis may occur in a relevant subset. While most cases of FCM-induced hypophosphatemia in our cohort appeared to be asymptomatic and transient, the clinical impact of hypophosphatemia in LTRs warrants consideration. The nonspecific symptoms of hypophosphatemia, including fatigue, muscle weakness, and bone pain, overlap substantially with common post-transplant complaints and symptoms of iron deficiency anemia itself. This overlap may lead to underrecognition of symptomatic hypophosphatemia in clinical practice. Although we did not observe serious complications such as fractures or osteomalacia in our cohort, these severe outcomes have been reported in patients receiving repeated FCM infusions. LTRs may be particularly vulnerable to the skeletal effects of prolonged hypophosphatemia given their high prevalence of bone disease, chronic corticosteroid use, and vitamin D deficiency. The potential for cumulative bone and mineral metabolism derangements with repeated FCM dosing underscores the importance of phosphate monitoring and consideration of alternative iron formulations in this population.

The analysis of potential predictors of hypophosphatemia following FCM infusion revealed a clear dose-dependent effect. The risk of developing hypophosphatemia increases significantly when 1000 mg of FCM is administered compared to 500 mg. Notably, the estimated effect became stronger after adjustment for baseline phosphate levels. Patients receiving 1000 mg FCM had slightly higher baseline phosphate concentrations than those receiving 500 mg FCM, which could partially mask the true dose effect. The model adjusted for baseline phosphate concentration suggests that the increased risk associated with the 1000 mg dose is independent of baseline phosphate status and not merely explained by patients being closer to the hypophosphatemia threshold before treatment. These findings underscore the role of FCM dose as a key modifiable risk factor and support consideration of lower FCM doses for the treatment of iron deficiency in LTRs. Thus, these findings support considering the strategy of using lower FCM doses to treat iron deficiency in LTRs. Moreover, as lower baseline phosphate level was a strong predictor for post-infusion hypophosphatemia, this finding highlights the potential clinical value of pre-treatment phosphate screening to guide iron dosing decisions.

Other variables, including baseline ferritin, creatinine, and vitamin D supplementation did not significantly influence hypophosphatemia risk.

While the use of tacrolimus compared to cyclosporine was associated with a more than two-fold increased odds (OR 2.15), this trend did not reach statistical significance (*p* = 0.126), warranting further investigation in larger cohorts. This potential association is supported by animal studies, in which tacrolimus has been shown to downregulate the sodium-phosphate cotransporter 2a (NPT2a), reducing renal phosphate reabsorption and thereby promoting hypophosphatemia [17, 19]. Although our data did not demonstrate a statistically significant association, the biological plausibility and observed trend suggest tacrolimus may contribute to phosphate loss following FCM infusion, particularly in patients with additional risk factors.

Our study has several limitations. The retrospective, single-center design without a control group (e.g., LTRs not receiving FCM or treated with alternative iron formulations such as ferric derisomaltose) limits causal inference. While we observed a high incidence of hypophosphatemia following FCM, we cannot definitively exclude other contributing factors specific to the post-transplant state. Additionally, we did not systematically assess clinical symptoms attributable to hypophosphatemia, and the retrospective design may have led to underreporting of nonspecific symptoms such as fatigue or muscle weakness. The absence of systematic FGF–23 measurements limits our ability to confirm the mechanistic pathway of hypophosphatemia in this population. Furthermore, some potentially relevant determinants of phosphate homeostasis, including PTH and TSH, were only available in a subset of patients and therefore could not be included in the multivariable analyses. Consequently, residual confounding by unmeasured disturbances in mineral and endocrine metabolism cannot be excluded. Finally, although 16 patients received more than one FCM infusion during the study period, this number was too small to allow a meaningful assessment of the cumulative effect of repeated FCM exposure on the risk, severity, or persistence of hypophosphatemia. This may be relevant given the increased risk of persistent hypophosphatemia and osteomalacia reported with repeated FCM dosing. Nonetheless, to our knowledge, this is the first study systematically evaluating FCM-associated hypophosphatemia in LTRs, a population uniquely predisposed to disturbances in mineral metabolism due to immunosuppressive therapy and chronic comorbidities.

Given the high incidence of hypophosphatemia observed in our cohort, particularly with higher FCM doses, clinicians should consider alternative intravenous iron formulations with lower hypophosphatemia risk when treating iron deficiency in LTRs. Ferric derisomaltose (also known as iron isomaltoside) has demonstrated significantly lower rates of hypophosphatemia compared to FCM in randomized controlled trials, with incidence rates of approximately 8% versus 74% [10]. Two large randomized trials (PHOSPHARE-IDA and PHOSPHARE-IBD) showed that ferric derisomaltose resulted in hypophosphatemia rates of only 7.9–8.1% compared to 73.7–75.0% with FCM, with no cases of severe hypophosphatemia (serum phosphate ≤1.0 mg/dL) in the ferric derisomaltose group versus 11.3% with FCM [10, 18]. When FCM is used, lower doses (500 mg rather than 1000 mg) should be preferred, and baseline phosphate screening should be performed to identify patients at higher risk. For patients requiring repeated iron infusions or those with baseline hypophosphatemia, ferric derisomaltose represents a safer alternative that maintains equivalent efficacy in correcting iron deficiency while substantially reducing the risk of phosphate disturbances [10, 20].

## Conclusion

This study demonstrated a high incidence of hypophosphatemia following FCM administration in lung transplant recipients, particularly after higher doses. Clinicians should be aware of this potential complication and consider phosphate monitoring, individualized dosing strategies, and alternative iron formulations in patients at increased risk. Prospective controlled studies are needed to better define the clinical relevance and optimal management of FCM-associated hypophosphatemia in solid organ transplant recipients.

## Data Availability

The datasets used and/or analyzed during the current study are available from the corresponding author on reasonable request.

## Abbreviations

CI: Confidence interval
CKD: Chronic kidney disease
CNI: Calcineurin inhibitor
COPD: Chronic obstructive lung disease
FCM: Ferric carboxymaltose
FDI: Ferric derisomaltose
FGF-23: Fibroblast growth factor 23
FU: Follow-up
GFR: Glomerular filtration rate
IV: Intravenous
LTRs: Lung transplant recipients
MCH: Mean corpuscular hemoglobin
MCV: Mean corpuscular volume
MMF: Mycophenolate mofetil
MPA: Mycophenolic acid
Mtor: Mechanistic target of rapamycin
NPT2a: Sodium-phosphate cotransporter 2a
OR: Odds ratio
PTH: Parathyroid hormone
SD: Standard deviation
SHPT: Secondary hyperparathyroidism

## References

[1] Steck D, Mostofi N, Tillinghast K, Wu D, Scovotti J, Cheng D et al (2023) The incidence of hypophosphatemia after lung transplant surgery: preliminary data from two major centers. Chest 164(4):A6457

[2] Schaefer B, Tobiasch M, Wagner S, Glodny B, Tilg H, Wolf M et al (2022) Hypophosphatemia after intravenous iron therapy: comprehensive review of clinical findings and recommendations for management. Bone 154:116202 10.1016/j.bone.2021.116202 34534708

[3] Wolf M, Weir MR, Kopyt N, Mannon RB, Von Visger J, Deng H et al (2016) A prospective cohort study of mineral metabolism after kidney transplantation. Transplant 100(1):184–193 10.1097/TP.0000000000000823 PMC4683035 26177089

[4] Baia LC, Heilberg IP, Navis G, de Borst MH (2015) Phosphate and FGF-23 homeostasis after kidney transplantation. Nature Reviews Nephrol 11:656–666 10.1038/nrneph.2015.153 26416497

[5] Bugge TB, Perch M, Rezahosseini O, Crone CG, Jensen K, Schultz HH et al (2022) Post-transplantation anemia and risk of death following lung transplantation. Transplant Proc 54(8):2329–2336 10.1016/j.transproceed.2022.07.012 36127173

[6] Lyseng-Williamson KA, Keating GM (2009) Ferric carboxymaltose: a review of its use in iron-deficiency anaemia. Drugs 69(6):739–756 10.2165/00003495-200969060-00007 19405553

[7] Rosano G, Schiefke I, Göhring U-M, Fabien V, Bonassi S, Stein J (2020) A pooled analysis of serum phosphate measurements and potential hypophosphataemia events in 45 interventional trials with ferric carboxymaltose. J Clin Med 9(11):3587 10.3390/jcm9113587 PMC7694774 33172157

[8] Schaefer B, Tobiasch M, Viveiros A, Tilg H, Kennedy NA, Wolf M et al (2021) Hypophosphataemia after treatment of iron deficiency with intravenous ferric carboxymaltose or iron isomaltoside—a systematic review and meta-analysis. Br J Clin Pharmacol 87(5):2256–2273 10.1111/bcp.14643 PMC8247006 33188534

[9] Strubbe M, David K, Peene B, Eeckhout B, Van der Schueren B, Decallonne B et al (2025) No longer to be ignored: hypophosphatemia following intravenous iron administration. Rev Endocr Metab Disord 26(1):125–135 10.1007/s11154-024-09926-5 39648248

[10] Wolf M, Rubin J, Achebe M, Econs MJ, Peacock M, Imel EA et al (2020) Effects of Iron Isomaltoside vs Ferric Carboxymaltose on Hypophosphatemia in Iron-Deficiency Anemia: Two Randomized Clinical Trials. JAMA 323(5):432–443 10.1001/jama.2019.22450 PMC7042864 32016310

[11] Pasricha SR, Tye-Din J, Muckenthaler MU, Swinkels DW (2021) Iron deficiency. Lancet 397(10270):233–248 10.1016/S0140-6736(20)32594-0 33285139

[12] Schaefer B, Wurtinger P, Finkenstedt A, Braithwaite V, Viveiros A, Effenberger M et al (2016) Choice of high-dose intravenous iron preparation determines hypophosphatemia risk. PLoS ONE 11(12):e0167146 10.1371/journal.pone.0167146 PMC5131956 27907058

[13] Schouten BJ, Hunt PJ, Livesey JH, Frampton CM, Soule SG (2009) FGF23 elevation and hypophosphatemia after intravenous iron polymaltose: a prospective study. J Clin Endocrinol Metab 94(7):2332–2337 10.1210/jc.2008-2396 19366850

[14] Hardy S, Vandemergel X (2015) Intravenous iron administration and hypophosphatemia in clinical practice. Int J Rheumatol 2015:468675 10.1155/2015/468675 PMC4426778 26000018

[15] Sari V, Atiqi R, Hoorn EJ, Heijboer AC, van Gelder T, Hesselink DA (2017) Ferric carboxymaltose-induced hypophosphataemia after kidney transplantation. Neth J Med 75(2):65–73 28276325

[16] Santulario-Verdú L, Echeverry-Navarrete E, Díez-López C, Canedo-Castelo M, Juvany-Roig R, García-Romero E et al (2022) Hypophosphatemia after intravenous ferric carboxymaltose in heart transplant patients. J Heart Lung Transplant 41(4):S220–S221

[17] Mohebbi N, Mihailova M, Wagner CA (2009) The calcineurin inhibitor FK506 (tacrolimus) is associated with transient metabolic acidosis and altered expression of renal acid-base transport proteins. Am J Physiol Renal Physiol 297(2):F499-509 10.1152/ajprenal.90489.2008 19439519

[18] Fragkos KC, Sehgal V, Rogers J, Arulrajan S, Pavanerathan P, Barragry J et al (2020) Hypophosphataemia after intravenous iron therapy with ferric carboxymaltose—real world experience from a tertiary centre in the UK. Gastroenterol Hepatol 2(5):205–214

[19] Moz Y, Levi R, Lavi-Moshayoff V, Cox KB, Molkentin JD, Silver J et al (2004) Calcineurin Abeta is central to the expression of the renal type II Na/Pi co-transporter gene and to the regulation of renal phosphate transport. J Am Soc Nephrol 15(12):2972–2980 10.1097/01.ASN.0000144207.44469.BE 15579499

[20] Dominguez Rieg JA, Domenech Acevedo M, Nogueira Coelho J, Stevens M, Thomas L, Rieg T (2025) Distinct roles of ferric carboxymaltose and ferric derisomaltose on phosphate homeostasis in iron deficiency anemia. Eur J Pharm Sci 214:107265 10.1016/j.ejps.2025.107265 PMC12924155 40935025

